# Dynamic Changes of the Phosphoproteome in Postmortem Mouse Brains

**DOI:** 10.1371/journal.pone.0021405

**Published:** 2011-06-22

**Authors:** Tsutomu Oka, Kazuhiko Tagawa, Hikaru Ito, Hitoshi Okazawa

**Affiliations:** Department of Neuropathology, Medical Research Institute, Tokyo Medical and Dental University, Tokyo, Japan; McGill University, Canada

## Abstract

Protein phosphorylation is deeply involved in the pathological mechanism of various neurodegenerative disorders. However, in human pathological samples, phosphorylation can be modified during preservation by postmortem factors such as time and temperature. Postmortem changes may also differ among proteins. Unfortunately, there is no comprehensive database that could support the analysis of protein phosphorylation in human brain samples from the standpoint of postmortem changes. As a first step toward addressing the issue, we performed phosphoproteome analysis with brain tissue dissected from mouse bodies preserved under different conditions. Quantitative whole proteome mass analysis showed surprisingly diverse postmortem changes in phosphoproteins that were dependent on temperature, time and protein species. Twelve hrs postmortem was a critical time point for preservation at room temperature. At 4°C, after the body was cooled down, most phosphoproteins were stable for 72 hrs. At either temperature, increase greater than 2-fold was exceptional during this interval. We found several standard proteins by which we can calculate the postmortem time at room temperature. The information obtained in this study will be indispensable for evaluating experimental data with human as well as mouse brain samples.

## Introduction

Protein phosphorylation has been implicated widely in the pathological mechanisms of neurodegenerative disorders including Alzheimer's disease (AD), frontotemporal dementia (FTD), dementia with Lewy bodies (DLB), Parkinson's disease dementia (PDD), and Huntington's disease (HD). For instance, hyperphosphorylated forms of tau have been identified as a major component of paired helical filament (PHF) [1], the common pathological hallmark of AD and tau-associated FTD. Hyperphosphorylation impairs the microtubule binding of tau and destabilizes microtubules. A process mediated by various serine/threonine kinases such as GSK3beta, PKA, Cdk5 and casein kinase II might also accelerate aggregation of tau into PHF [2]–[5]. In the DLB brain, phosphorylated alpha-synuclein at Ser 129 has been detected by mass spectrometry and phosphorylation-accelerated aggregation of alpha-synuclein has been shown *in vitro* [6]. The increase of toxicity by phosphorylation was also suspected in a Drosophila model of PDD [7].

However, the significance of variable phosphorylation levels of inclusion body component proteins remains controversial. For instance, in alpha-synuclein, phosphorylated tyrosine and serine residues seem to have opposite effects on cellular toxicity [8]. In addition, the role of phosphorylation can differ among neurodegenerative diseases. In contrast to the cases of tau and synuclein, phosphorylation of mutant huntingtin (Htt) at Ser 421 in response to IGF treatment reduced toxicity [9]; similarly, phosphorylation of Htt at Ser 13 and 16 alleviated phenotype in a mouse model of HD [10].

Furthermore, one can easily imagine that a number of phosphoproteins other than aggregated proteins would quantitatively change in the context of neurodegenerative disorders. It is possible that such phosphorylation also affects neurodegeneration. Therefore, in order to understand the whole scheme of pathological functions of protein phosphorylation, it is necessary to perform a full “omics” analysis of phosphoproteins in human brains.

However, the most significant obstacle to this approach is posed by the preservation conditions of human brains. There is no world standard that specifies how to preserve postmortem human bodies before the brains are cut, frozen and kept in the brain bank. After patients' deaths, their bodies are left on their beds at room temperature for different length of time before being transferred to the morgue in the pathology department. The time between brain sectioning and freezing on dry ice also varies among samples and among institutions.

Therefore, we need to know how the phosphoproteome changes in postmortem brains during the process of preservation. Based on such knowledge, we could potentially design a specific preservation protocol for brain banks to follow in order to enable reproducible phosphoproteome analysis in human samples. Even with such a standard, specific methods for adjusting phosphoproteomic data based on time and temperature would also be required. For these purposes, we performed proteome-wide analyses of phosphoproteins on mouse brains that had been kept at room temperature or 4°C for different lengths of time. The results reveal surprisingly diverse patterns of chronological changes of phosphoproteins. Data such as ours will be crucial in interpreting the phosphoprotein data obtained to date, as well as future data, from human brain samples.

## Results

### Diverse changes of postmortem phosphoproteins

To investigate the effect of postmortem time on phosphoproteins before the brain was isolated and frozen, we kept 12 week-old C57BL/6J mice at 25°C or 4°C for a different length of time (0, 3, 12, 72 hrs) after they were sacrificed by deep euthanasia. After the incubation period, the mice were dissected and their brains immediately frozen in liquid nitrogen. The brain samples were thawed at 4°C in T-PER Tissue Protein Extraction Reagent and immediately used for sample preparation, as described in Methods. The quantitative whole proteome mass analysis was performed with Q-STAR (AB SCIEX) and repeated for three sets of samples. Each set included cerebral cortex samples from a mouse body kept at 25°C or 4°C for different lengths of time (0, 3, 12, 72 hrs).

**Figure 1:**
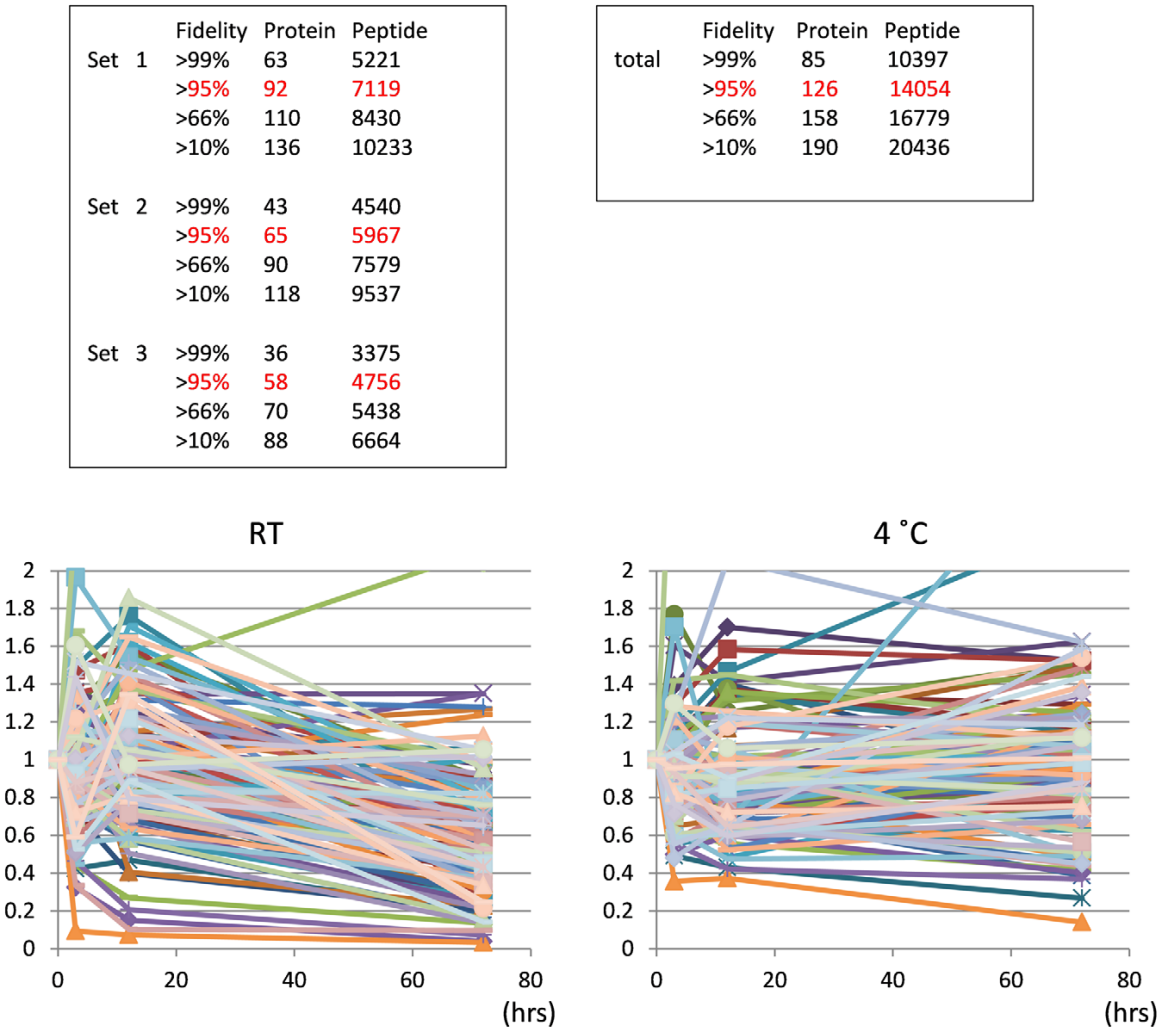
Postmortem dynamics of phosphoproteins revealed by quantitative mass analysis. The numbers of proteins and peptides identified from three sets of mass analysis are listed. 126 proteins were identified with more than 95% fidelity from the merged data of three sets. The lower graphs show the chronological change of phosphoproteins during preservation at 25°C and 4°C.

Relative quantity compared to the initial value at 0 hr was obtained for each phosophoprotein, and the mean value among three sets was calculated for each time point and temperature (Figure 1). Surprisingly, the postmortem changes in phosphoproteins showed extreme diversity (Figure 1). When increase and decrease were defined as more than 1.2 and less than 0.8 fold, respectively, 26% of total phosphoproteins decreased rapidly from 0 to 3 hrs, while 17% of proteins increased during this interval at room temperature (25°C). 75% of total phosphoproteins were reduced after more than 12 hrs at room temperature (25°C), but some exceptional phosphoproteins (5.5%) continued to increase from 12 to 72 hrs. In contrast to the pattern at room temperature, 55% of phosphoproteins were relatively stable after more than 12 hrs at 4°C. However, there were also exceptional cases whose levels increased or decreased rapidly.

### Categorization of temperature-dependent changes of phosphoproteins

**Figure 2:**
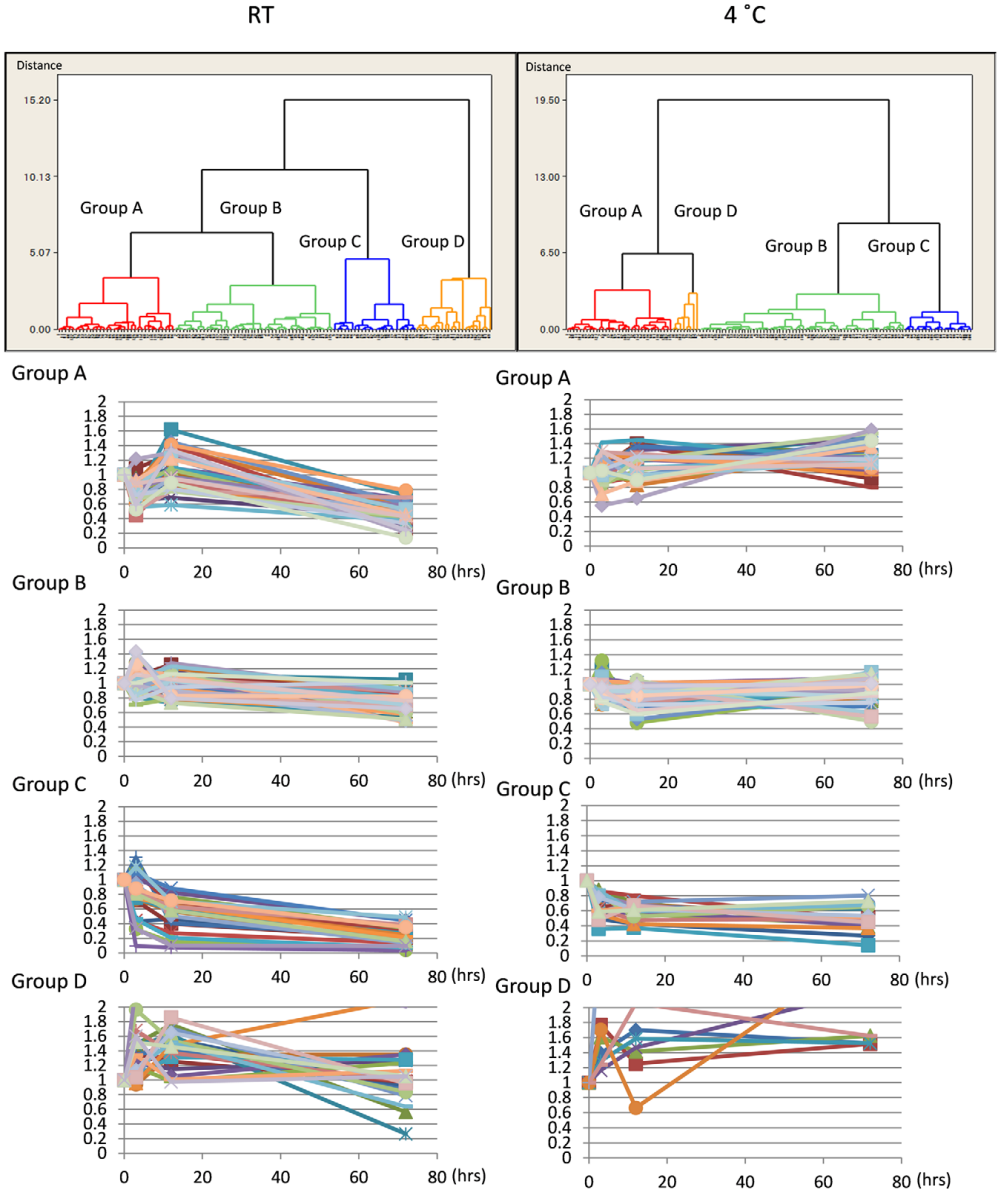
Cluster analysis of postmortem phosphoprotein dynamics. Dendrograms of total identified proteins at RT (25°C) and 4°C were drawn by cluster analysis. Cerebral cortex phosphoproteins were classified into four groups according to the changing pattern (upper panels). At 25°C, 34, 46, 24 and 22 proteins belong to Groups A, B, C and D, respectively. At 4°C, 33, 64, 21 and 8 proteins belong to Groups A, B, C and D, respectively. Lower graphs show the patterns of chronological change for phosphoproteins in each group.

We suspected that a mixture of different patterns might cause the extreme diversity of chronological changes. Therefore, we categorized the data by cluster analysis. At each temperature, phosphoproteins were classified into four groups (Figure 2). Group A−C showed typical patterns at each temperature, while group D (“miscellaneous”) contained a wide variety of behaviors. At room temperature, Group A exhibited a transient increase and decline after 12 hrs. Group B exhibited a relatively constant and slow decrease until 72 hrs. Group C exhibited a rapid decrease until 12 hrs and a more gradual decrease after 12 hrs. At 4°C, Group A tended to increase slowly; group B was almost stable until 72 hrs; and group C decreased rapidly until 3 hrs and then stabilized.

**Figure 3:**
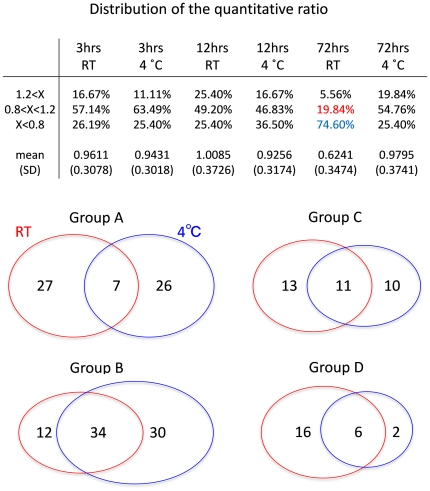
Comparison between RT and 4°C groups. (A) Identified phosphoproteins at each time point were classified into three groups based on the relative quantity to the initial value at 0 hr. (B) Corresponding groups are compared between RT (25°C) and 4°C to evaluate the similarity between groups.

The classification revealed that in each temperature group, the initial changes in phosphoprotein levels followed diverse paths until 12 hrs. Later than 12 hrs, all phosphoproteins either stabilized or gradually decreased at room temperature. At the same time, there were exceptional proteins whose changes can hardly be expected. These patterns of change were also supported when proteins were classified by the ratio of changes (Figure 3). Until 12 hrs, the percentage of increased (X>1.2), unchanged (0.8<X<1.2) or decreased (X<0.8) phsophoproteins was almost the same between 25°C and 4°C. However, the percentage of decreased proteins (X<0.8) increased markedly at times later than 12 hrs at 25°C.

As shown in Figure 2, the pattern for Group B at 25°C looked similar to that of Group B at 4°C. This was also the case for Group C. This impression was supported by a comparison of phosphoproteins belonging to these groups (Figures S1, S2, S3). The comparison also indicated that expression of a majority of proteins was relatively constant, and that chronological change can be expected at both temperatures.

### Selection of standard phosphoproteins

**Figure 4:**
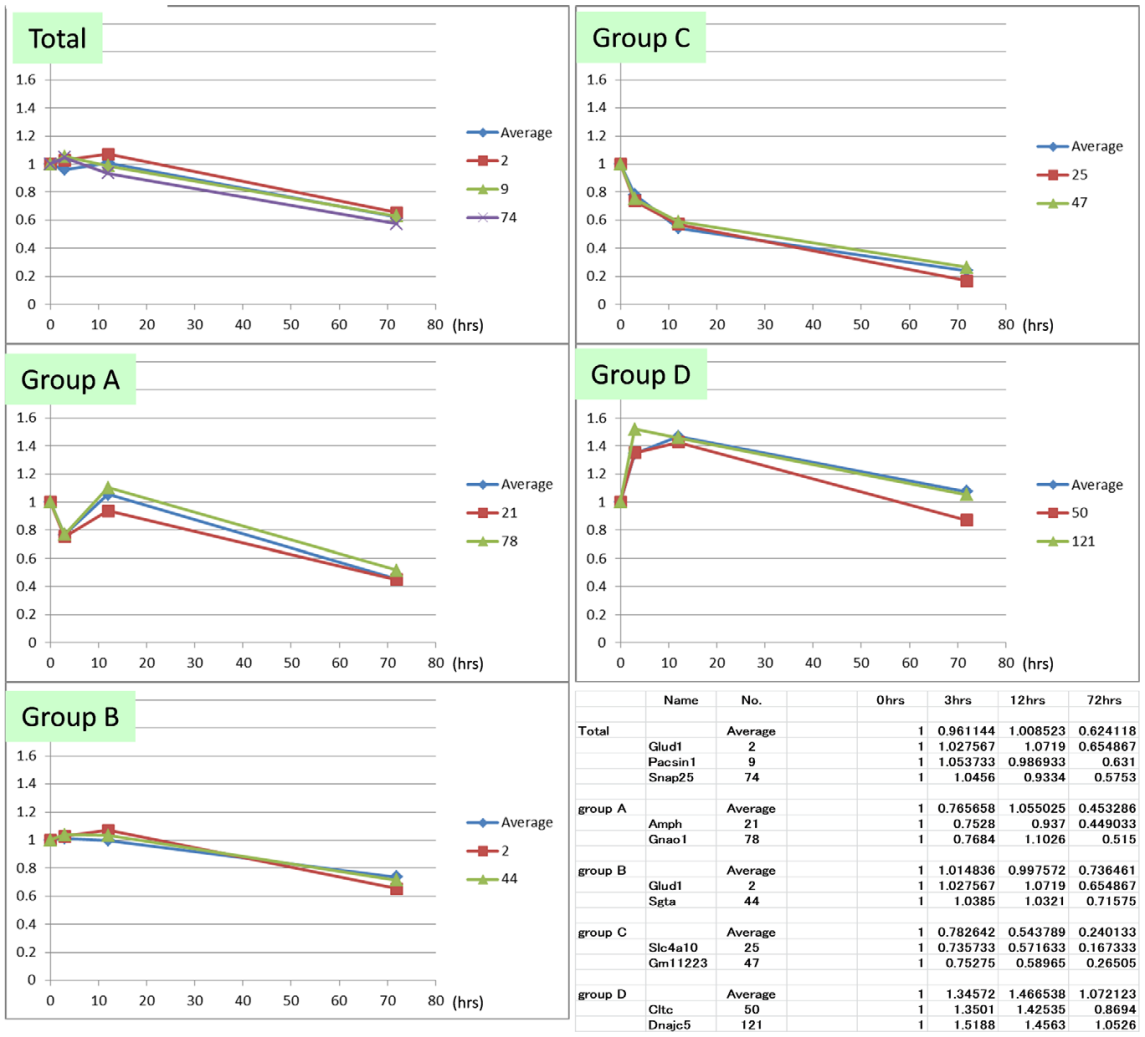
Representative genes of each clustered group at RT. Chronological changes at 25°C of the mean value of total identified phosphoproteins (Total) and of phosphoproteins belonging to each group (Groups A−D) are shown. Proteins most similar to the mean value pattern were also selected.

**Figure 5:**
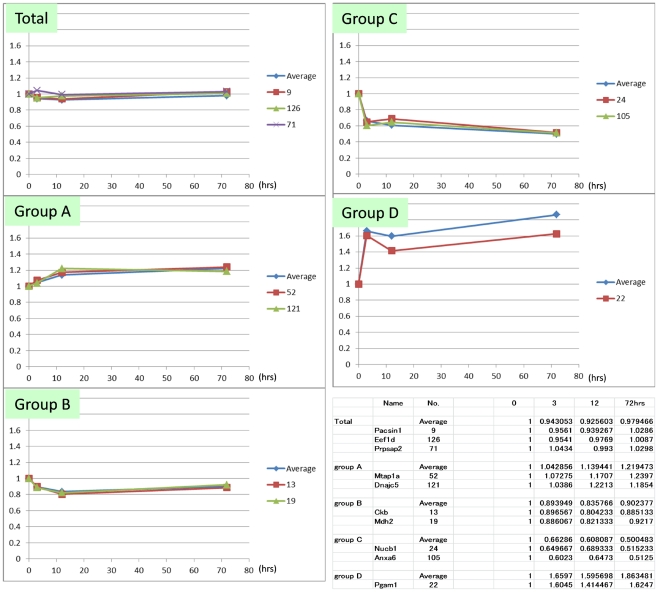
Representative genes of each clustered group at 4°C. Chronological changes at 4°C of the mean value of total identified phosphoproteins (Total) and of phosphoproteins belonging to each group (Groups A−D) are shown. Representative proteins that mimic the mean value pattern are also selected.

We next selected standard proteins that can be used for quality control of brain tissue even when information about postmortem processing conditions is not available. We first plotted the mean value of each group (Figures 4, 5). The mean value for total protein level declined at 0.006/hr after 12 hrs at 25°C (Figure 4); in contrast, at 4°C, the mean value was stable until 72 hrs (Figure 5). Corresponding to each group, we selected representative proteins that mimicked the pattern of mean values (Figures 4, 5). The standard proteins that represented the decline of total phosphoproteins at room temperature were Glud1, Pacsin1 and Snap25. Glud1 is highly expressed in the brain and is known to function as a mitochondrial enzyme converting glutamate to 2-oxoglutarate [11], thereby affecting the blood ammonia level [12]. Pacsin1, a neural isoform of Pacsin, is a cytoplasmic phosphoprotein involved in vesicle formation [13] and endocytosis regulation [14]. It also interacts with huntingtin at synapses [15]. Snap25 is a membrane-bound pre-synaptic protein and a component of the SNARE complex [16], which is essential for synaptic vesicle fusion [17] and Ca^2+^ response [18]. On the other hand, the standard proteins representing the stability of total phosphoproteins at 4°C were Pacsin1, Eefeld and Prpsap2. Eefeld delivers aminoacyl tRNAs to the ribosome [19], and is also known to catalyze the exchange of GDP bound to Elongation Factor 1α with GTP [20] which is stimulated by PKC [21]. Prpsap2 negatively regulates phosphoribosyl pyrophosphate (PRPP) synthetase [22]. These data together indicate that Pacsin1 is a good control protein to use in evaluation of the postmortem interval during which the body was left at room temperature before transfer to the morgue in the pathology department.

**Figure 6:**
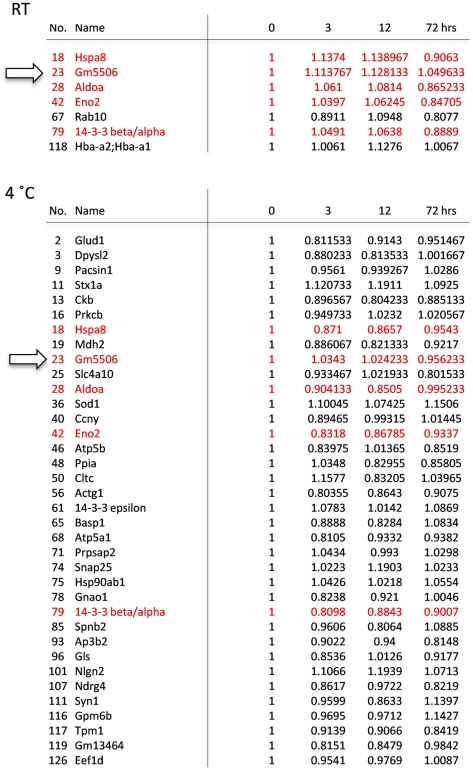
Stable proteins at RT and 4°C. Proteins whose relative amount stayed within 0.8 to 1.2 during 72 hrs are listed. 7 and 36 proteins matched this criterion at 25°C and 4°C, respectively. Proteins found at both temperatures are marked in red. Arrows indicate the standard protein, which is stable at RT (25°C) and 4°C.

**Figure 7:**
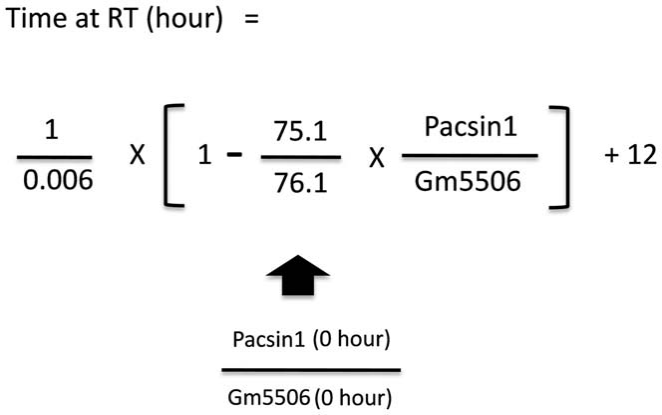
The formula for calculating the postmortem time at room temperature. The formula was based on the characters of Pacsin1 and Gm5506. Pacsin1 decreases at a constant rate at room temperature but it is stable at 4°C for 72 hrs. In contrast, Gm5506 is stable both at room temperature and 4°C. The ratio between Pacsin1 and Gm5506 at 0 hr (76.1∶75.1) is also known. If the ratio between Pacsin1 and Gm5506 at 0 hr in fresh human brain samples is known, we can calculate the postmortem time at RT in human brain with the formula.

We also found several phosphoproteins that were relatively stable up to 72 hrs at room temperature (Figure 6). Importantly, one of them, Gm5506 (α-enolase) was stable also at 4°C (Figure 6). As shown in Figures 4 and 5, the change of Pacsin1 exactly matches with the average change of total phosphoprotein (Figures 4, 5). Pacsin1 decreases at a constant rate at room temperature but it is stable at 4°C for 72 hrs. In contrast, Gm5506 is stable both at room temperature and 4°C. The ratio between the absolute signal values of Pacsin1 and Gm5506 at 0 hr (76.1∶75.1) is also known. From these values, we can calculate the postmortem time at room temperature according to the formula (Figure 7).

## Discussion

This study had three main goals: 1) to investigate how the phosphoproteome is changed in postmortem brains over the course of preservation; 2) to propose a specific preservation protocol that is suitable for phosphoproteome analysis of brain tissue; 3) to develop a specific method for adjusting phosphoprotein data based on time and temperature. For these purposes, we performed whole-proteome analyses of phosphoproteins using mouse brains that had been kept at room temperature or 4°C for different lengths of time.

First, we learned that the chronological changes in phosphoproteins are surprisingly diverse. Most proteins (82.5% at RT and 93.7% at 4°C) change following several typical patterns (Group A, B or C). However, the change of the other proteins is hardly prospected (Group D). This result suggests several things. If a phosphoprotein of interest belongs to the three regular groups (Groups A−C), we can evaluate whether the value obtained in an experiment is increased or decreased based on the pattern obtained in this study. If a target phosphoprotein in future research belongs to Group D, however, caution must be taken in evaluating its levels. Although the patterns are limited to 126 proteins in our current study, the database would be expanded to a larger number of phosphoproteins. We are now obtaining data with a higher grade of mass analysis; the results will be made available as an open database in the future.

As hyperphosphorylation of some specific proteins has been implicated as causative in neurodegenerative disorders, we asked whether our current results included such important proteins. However, APP [23]-[27], Presenilin1/2 [28], [29], tau [30], [31], Apolipoprotein E [32], Cdk5 [33], TDP-43 [34] or GSK-3beta [35], which are implicated in Alzheimer's disease and tauopathy, were not included in these results. Also, alpha-synuclein [36], [37], Leucine-rich repeat kinase 2 [38], [39], ubiquitin carboxyl-terminal hydrolase L1 [40], Parkin [41], Pink1 [42], Grb10-Interacting GYF Protein-2 [43], or Omi/HtrA2 [44], which are implicated in Parkinson's disease, are not included. The postmortem changes in these proteins will also become clear in a future study.

Although postmortem changes in phosphoproteins were diverse over the first 12 hrs, they were generally inclined to decrease from 12 to 72 hrs. Since only one protein was increased more than 2-fold during 72 hrs at room temperature, and only two proteins were increased more than 2-fold during 72 hrs at 4°C, increases more than 2-fold can be suspected as abnormal in any postmortem samples that were kept at 4°C within 72 hrs. However, after getting such a result, one should come back to postmortem omics data such as ours in order to check the validity of the result.

Second, we can now propose how the brain samples should be prepared for the analysis of phosphoproteins. Our results showed that some proteins rapidly decreased within 12 hrs at room temperature (Figure 2, Group C at RT) while some other proteins increased within 12 hrs at room temperature (Figure 2, Group A at RT). After 12 hrs, most phosphoproteins declined at a constant rate (0.006/ hr), and we learned that the mean value of phosphoproteins at room temperature was remarkably decreased at 72 hrs (Figure 3).

Even at 4°C, some proteins were increased or decreased within 12 hrs (Figure 2, Groups A−C at 4°C). Later than 12 hrs, however, the amounts of phosphoprotein were very stable (Figure 2, Groups A−C at 4°C). This probably means that it takes some time before the brain temperature reaches 4°C, and some phosphoproteins change quantitatively during the time. But after the brain temperature was brought to 4°C, phosphoproteins were stable until 72 hrs. Measurement of postmortem deep brain temperature supported this speculation (Figure S4).

Thus, the body should be chilled to 4°C as soon as possible, no later than 12 hrs after death. In other words, the brain temperature should become 4°C within 12 hrs postmortem. Even with more or less immediate chilling, however, one should refer to our database in this study (Figure S5) showing how each phosphoprotein changes over the course of the first 12 hrs. We will update the database, covering far more phosphoproteins, in the near future.

Third, we propose a method for estimating the postmortem time spent at room temperature. Pacsin1 decreases at a constant rate at room temperature, exactly matching with the average change of total phosphoprotein (Figures 4, 5). In addition, Pacsin1 is stable at 4°C for 72 hrs. In contrast, Gm5506 is stable both at room temperature and 4°C. The absolute signal value ratio between Pacsin1 and Gm5506 at 0 hr (76.1∶75.1) is also known. Therefore, we could calculate the postmortem time at room temperature from these values (Figure 7). We would be able to use Eefeld and Prpsap2, as well as Hspa8/Hsp70-4 or 14-3-3 α/β, as other standards for the same purpose. If we can obtain a fresh human brain sample and measure the absolute ratio between Pacsin1 and Gm5506/α-enolase at 0 hr, this method can be used to calculate the postmortem time in humans. However, for ethical reasons, we have not obtained such a value currently.

This study left several issues for future studies. First, we may have to add earlier time points to detect certain unexpected changes of phosphoproteins. Second, we need to analyze other parts of the brain like brainstem, cerebellum, and spinal cord. The dynamics of phosphoproteins in such brain parts might be different from that of cerebral cortex. The information will be necessary for the phosphoprotein in human diseases like spinocerebellar ataxia or multiple system atrophy.

In conclusion, our data has provided crucial information that can be used to interpret future data, as well as previously reported experimental data regarding phosphoproteins. We may have to re-evaluate previous reports using human samples from the standpoint of postmortem time and temperature. Future experiments using human samples should definitely be evaluated using our data, which will be expanded and delivered as an open database.

## Materials and Methods

### Ethics statement

All procedures were approved by the Institutional Animal Care and Use Committee of the Tokyo Medical and Dental University (MR:2010-002) and performed according to the guidelines of Ministry of Education, Culture, Sports, Science and Technology (MEXT) of Japanese government.

### Sample preparation

12 week-old C57BL/6J mice were kept at 25°C or 4°C for different lengths of time (0, 3, 12, 72 hrs) after they were sacrificed by deep euthanasia using ethyl ether. Mice were put in a glass bottle (5L) in which the air was saturated by ethyl ether. The mice were deeply anesthetized by ether vapor at three minutes later when they were sacrificed. Their cerebral cortex was isolated, immediately frozen in liquid nitrogen, and stored at −80°C. Protein was extracted from the cerebral cortex using the T-PER Tissue Protein Extraction Reagent (Thermo Fisher Scientific Inc., USA). The amount of the protein was measured using the BCA Protein Assay Reagent (Thermo Fisher Scientific Inc.).

### iTRAQ labeling

Samples containing 95 µg of protein were denatured in 0.1% SDS, and reduced in 5 mM TCEP (tris-2-carboxyethyl phosphine) for 1 hr at 60°C. Cysteine residues were blocked with 10 mM MMTS (methyl methanethiosulfonate) for 10 min at 25°C, and then samples were digested with trypsin (10∶1 protein/enzyme w/w) for 24 hrs at 37°C. The digested proteins (peptides) were then passed through a Sep-Pak Light C18 cartridge column (Waters Corporation, USA) to be desalted. Phosphopeptides were enriched using the Titansphere Phos-Tio Kit (GL Sciences Inc., Japan), and desalted again with a Sep-Pak Light C18 cartridge column. The peptides in each individual sample were labeled separately using the iTRAQ Reagent-multiplex assay kit (AB SCIEX Ins.) for 2 hrs at 25°C. The labeled peptide pools were then mixed together. Next, the peptide mixture was subjected to SCX (Strong Cation Exchange) chromatography (AB SCIEX Ins.). The peptide mixture was eluted in a stepwise gradient from 20, 60, 100, 150, 200, and 350 mM KCl in 10 mM KH_2_PO_4_ (pH 3.0), 25% acetonitrile. The peptide fractions were dried and desalted using a Sep-Pak Light C18 cartridge column.

### Quantitative whole proteome mass analysis

The dried peptide fractions were re-suspended in 2% acetonitrile and 0.1% formic acid. Each SCX fraction was analyzed using a DiNa Nano-flow LC system (KYA Technologies Corporation, Japan) and Q-STAR® Elite Hybrid LC/MS/MS System (AB SCIEX Ins.). The samples were loaded onto a 0.1 mm×100 mm C18 column and eluted with a gradient of 5−100% solution B (80% acetonitrile and 0.1% formic acid) in solution A (2% acetonitrile and 0.1% formic acid). The flow rate was 300 nL/min, and ion spray voltage was 1.8 kV. The IDA (Information Dependent Acquisition) setting was 400−1800 *m/z* with two to four charges. Analyst QS 2.0 software (AB SCIEX Ins.) was used to identify each peptide. Quantification of each peptide was based on TOF-MS electric current detected during the LC-separated peptide peak, adjusted by the charge/peptide ratio. Quantification of a protein was deduced by averaging the quantities of multiple peptide peaks from the protein. These processes were automatically performed using the Q-STAR® Elite Hybrid LC/MS/MS Hybrid System (AB SCIEX Ins.)

### Cluster analysis of identified proteins

The quantitative ratio between 0 hr and the other 6 time points (1, 25°C for 3 hrs; 2, 25°C for 12 hrs; 3, 25°C for 72 hrs; 4, 4°C for 3 hrs; 5, 4°C for 12 hrs; 6, 4°C for 72 hrs) in each detected protein was obtained using the Protein Pilot software (AB SCIEX Ins.) analysis. The chronological pattern was classified into four groups by clustering analysis using Minitab 16 software with the Ward method and Euclidean distance. Dendrograms were drawn using the same software.

## Supporting Information

Figure S1 Proteins belonging to each cluster group at 25°C are listed. Proteins that are members of the same groups at 25°C and 4°C are marked in red.
(TIF)

Figure S2 Proteins belonging to each cluster group at 4°C are listed. Proteins that are members of the same groups at 25°C and 4°C are marked in red.
(TIF)

Figure S3 Full names of the gene symbols, listed in alphabetical order.
(TIF)

Figure S4 Chronological change of the deep brain temperature in mouse body at 25°C or 4°C. Temperature was measured using a needle thermometer (Testo 905-T1, Japan) in mouse bodies left at 25°C or 4°C and mean +/− SD (n = 4) are shown in the graph.
(TIF)

Figure S5 The Excel file includes all the data on 126 proteins.
(TIF)
