# Supplementary figures and images for: Dynamic Changes of the Phosphoproteome in Postmortem Mouse Brains
Source: PLoS One. 2011 Jun 22;6(6):e21405. doi: 10.1371/journal.pone.0021405 (PMC3120861; doi:10.1371/journal.pone.0021405)

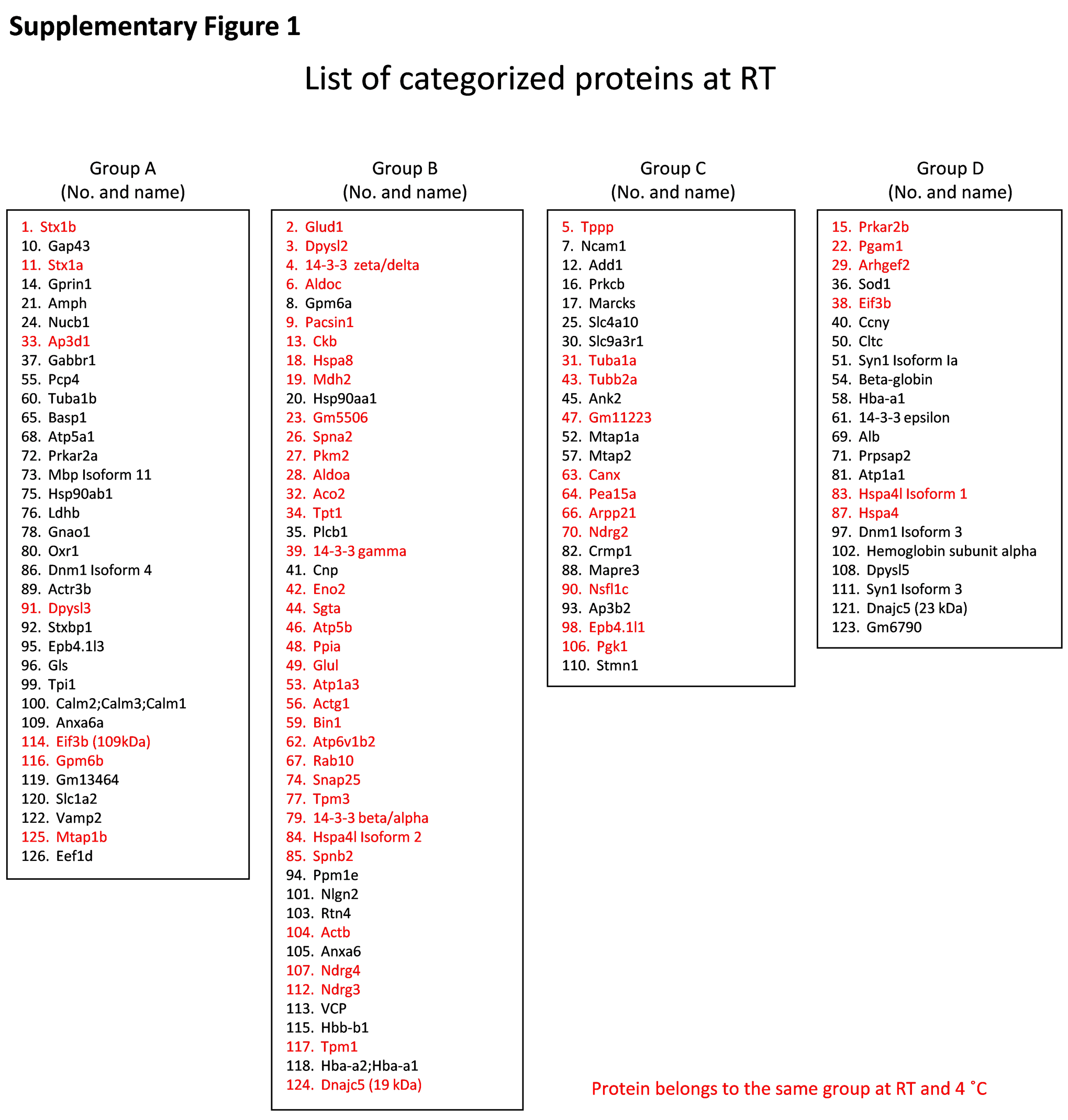

Supplement: Figure S1 — Proteins belonging to each cluster group at 25°C are listed. Proteins that are members of the same groups at 25°C and 4°C are marked in red. (TIF) [file pone.0021405.s001.tif]

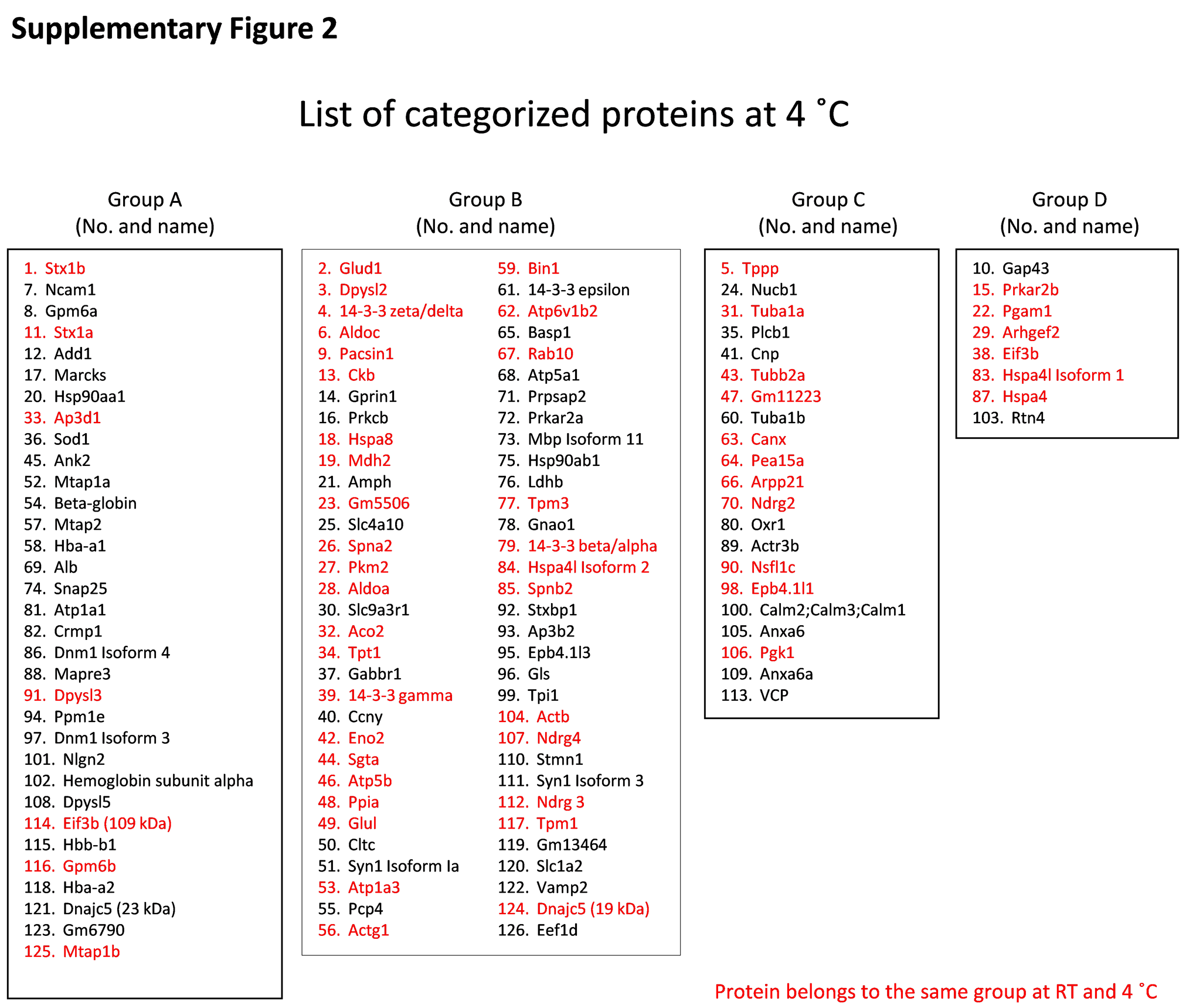

Supplement: Figure S2 — Proteins belonging to each cluster group at 4°C are listed. Proteins that are members of the same groups at 25°C and 4°C are marked in red. (TIF) [file pone.0021405.s002.tif]

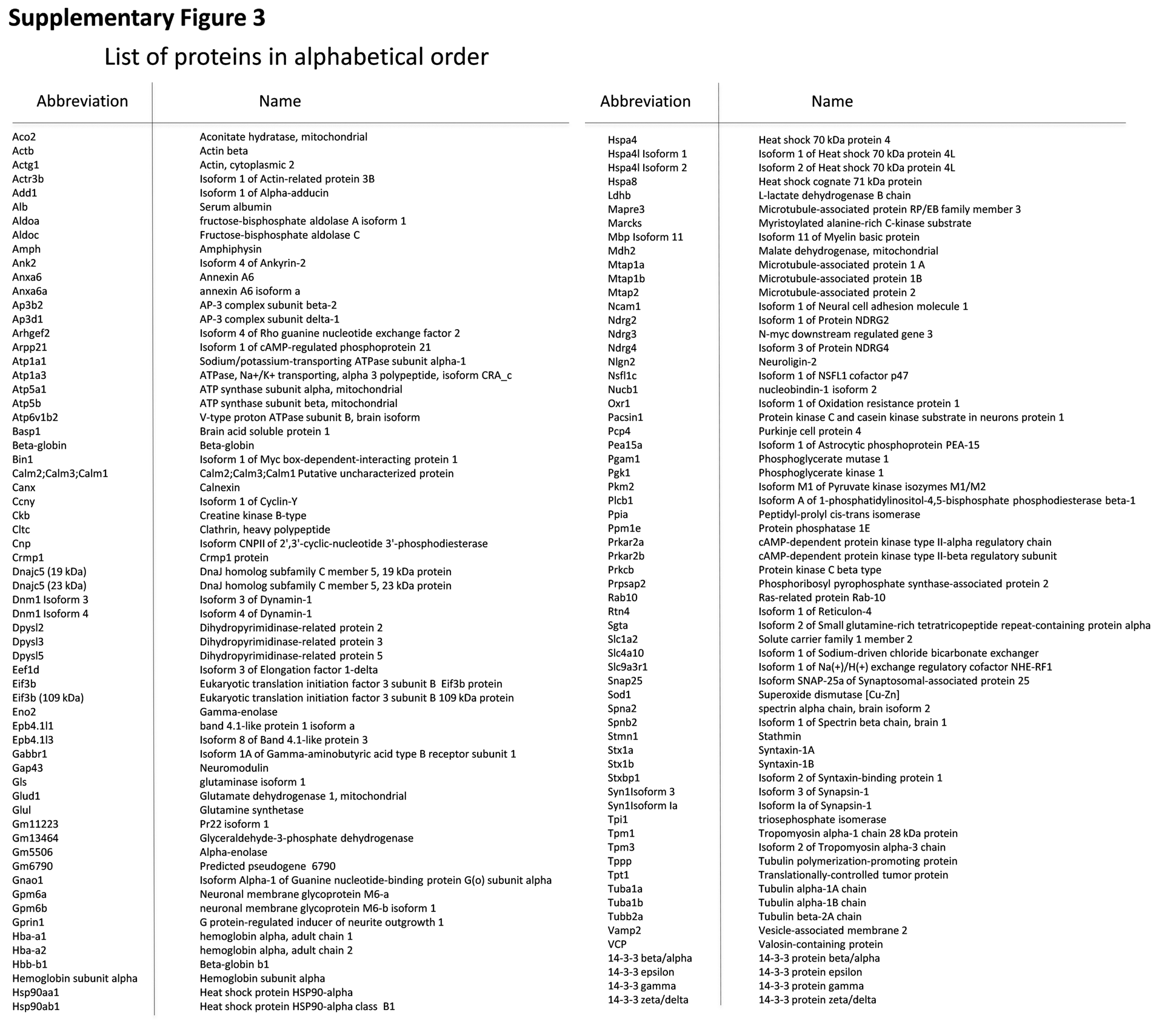

Supplement: Figure S3 — Full names of the gene symbols, listed in alphabetical order. (TIF) [file pone.0021405.s003.tif]

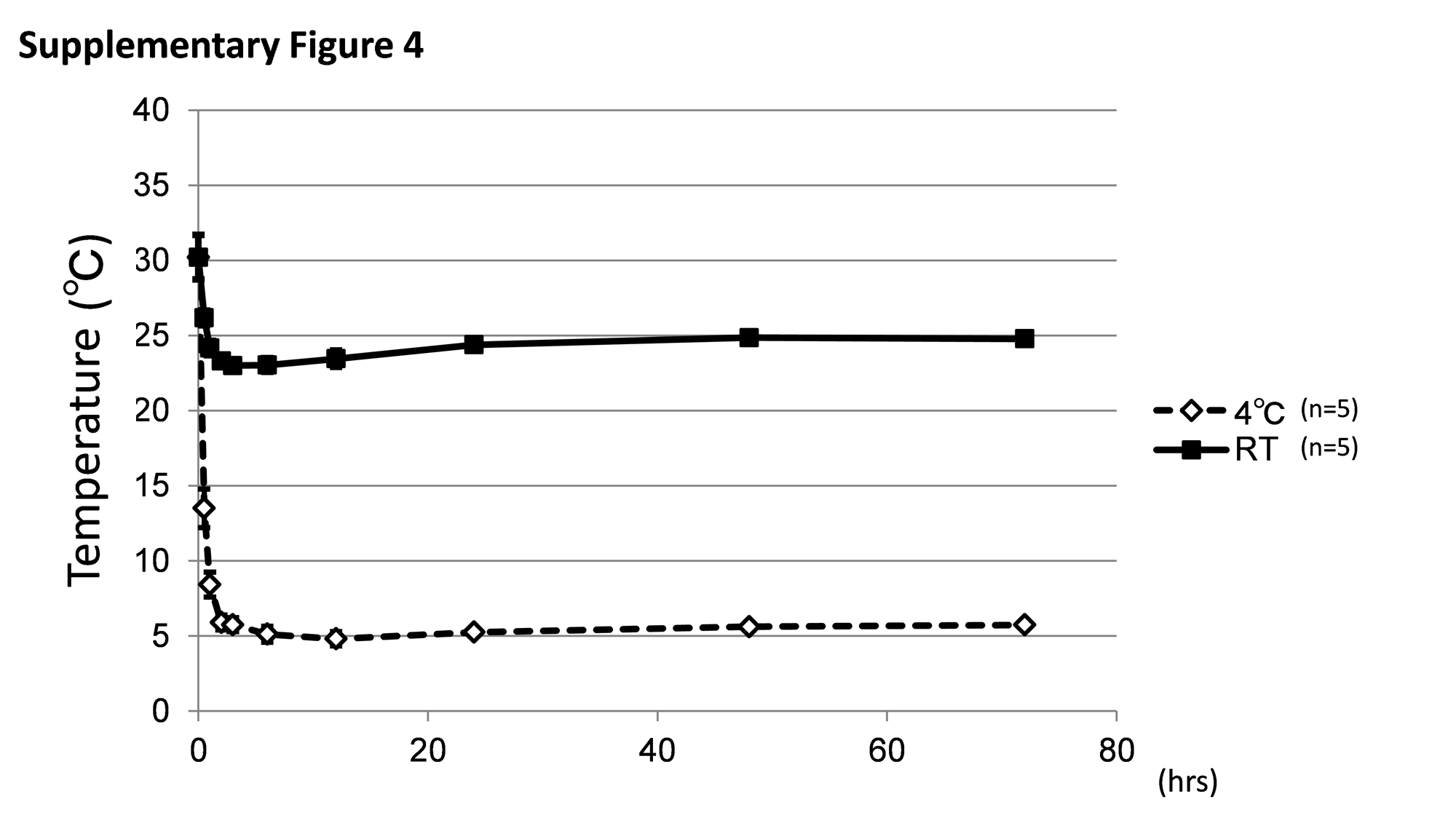

Supplement: Figure S4 — Chronological change of the deep brain temperature in mouse body at 25°C or 4°C. Temperature was measured using a needle thermometer (Testo 905-T1, Japan) in mouse bodies left at 25°C or 4°C and mean +/− SD (n = 4) are shown in the graph. (TIF) [file pone.0021405.s004.tif]

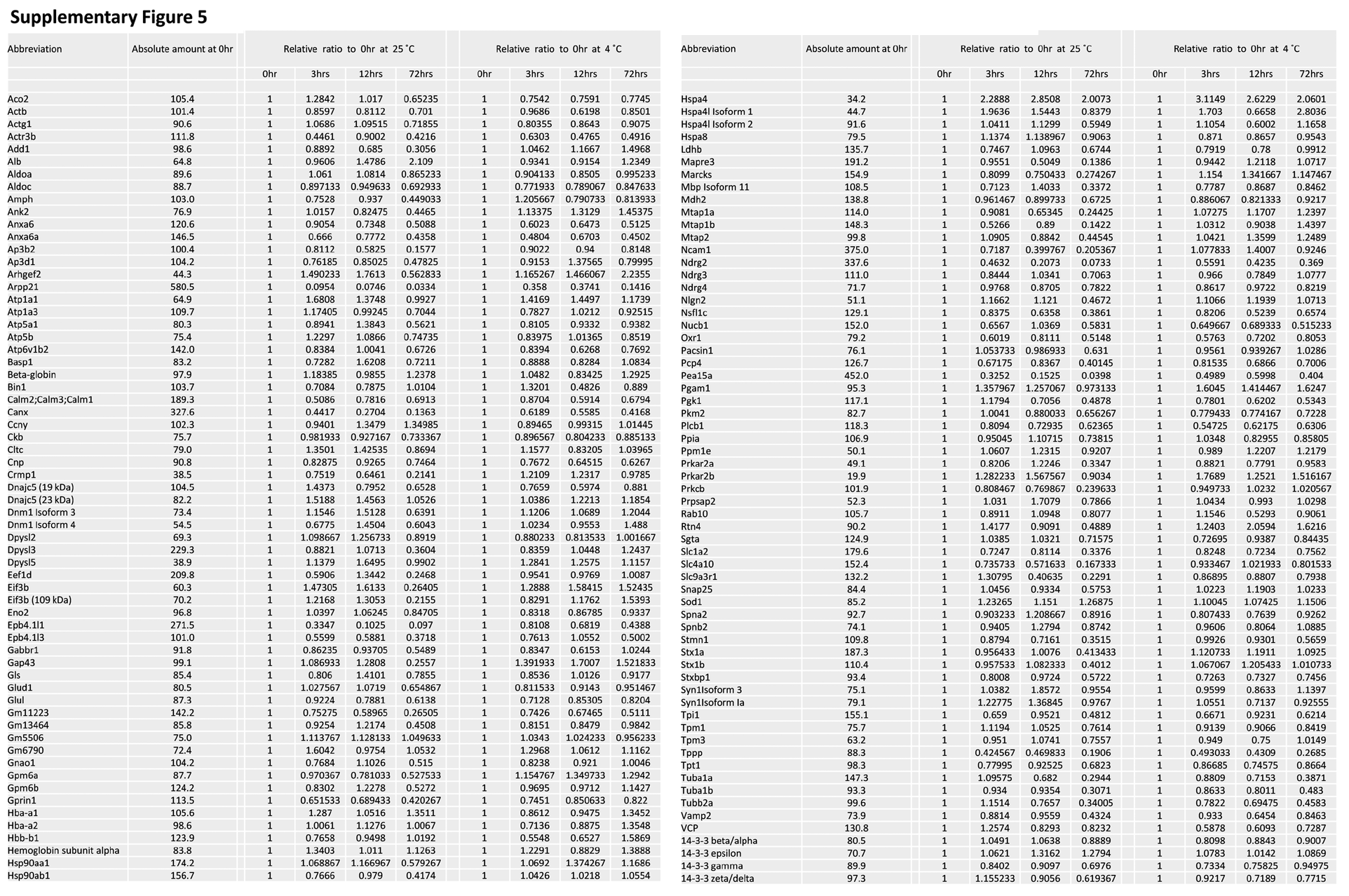

Supplement: Figure S5 — The Excel file includes all the data on 126 proteins. (TIF) [file pone.0021405.s005.tif]
